# Data on multimerization efficiency for short linear DNA templates and phosphoryl guanidine primers during isothermal amplification with Bst exo- DNA polymerase

**DOI:** 10.1016/j.dib.2020.105188

**Published:** 2020-01-25

**Authors:** Ravil R. Garafutdinov, Assol R. Sakhabutdinova, Maxim S. Kupryushkin, Dmitrii V. Pyshnyi

**Affiliations:** a Institute of Biochemistry and Genetics, Ufa Federal Research Center of the Russian Academy of Sciences, Russian Federation; b Institute of Chemical Biology and Fundamental Medicine, Siberian Branch of the Russian Academy of Sciences, Russian Federation

**Keywords:** Isothermal amplification, Multimerization, Bst exo- DNA polymerase, Phosphoryl guanidine oligonucleotides (PGO)

## Abstract

This article reports experimental data related to the research article entitled “Prevention of DNA multimerization using phosphoryl guanidine primers during isothermal amplification with Bst exo- DNA polymerase” (R.R. Garafutdinov, A.R. Sakhabutdinova, M.S. Kupryushkin, D.V. Pyshnyi, 2020) [1]. Here, multimerization efficiency in terms of Tt (time-to-threshold) values obtained for artificial DNA templates with the different nucleotide sequences during isothermal amplification with Bst exo- DNA polymerase is given. Data on the influence of phosphoryl guanidine primers (PGO) on multimerization for the LTc template which has shown high efficiency of multimerization are presented as well.

**Table:** Specifications Table

Subject	Biochemistry, Biotechnology, Molecular Biology
Specific subject area	Nucleic acids amplification, nucleic acids chemistry
Type of data	Tables
How data were acquired	DNA amplification in real-time mode using iQ5 thermal cycler (Bio-Rad Laboratories, USA)
Data format	Raw and analyzed
Parameters for data collection	Amplification experiments were performed under common conditions in real-time mode using Eva Green intercalating dye. DNA templates with different nucleotide sequences and corresponding natural and modified primers with one, two or three phosphoryl guanidine groups were used.
Description of data collection	Time-to-threshold (Tt) values were found from real-time amplification experiments.
Data source location	Institute of Biochemistry and Genetics Ufa Federal Research Center Russian Academy of Sciences, Ufa, Russia; Institute of Chemical Biology and Fundamental Medicine, Siberian Branch of the Russian Academy of Sciences, Novosibirsk, Russia
Data accessibility	Raw data are provided in Supplementary file. All other data is with this article.
Related research article	Ravil R. Garafutdinov, Assol R. Sakhabutdinova, Maxim S. Kupryushkin, Dmitrii V. Pyshnyi Prevention of DNA multimerization using phosphoryl guanidine primers during isothermal amplification with Bst exo- DNA polymerase Biochimie **168**, 2020, 259–267. DOI 10.1016/j.biochi.2019.11.013.

**Table:** 

**Value of the Data** • The data presented indicate that multimerization proceeds efficiently during isothermal amplification using Bst exo- polymerase and slightly depends on the nucleotide sequence of the DNA templates. • Three contiguous phosphoryl guanidine (PG) groups in the middle of the both primers are enough and the most appropriate for prevention of multimerization. • The obtained results allow to design of improved primers for isothermal amplification with Bst exo- polymerase that could provide accurate and reliable DNA diagnostics.

## 1 Data

DNA multimerization is a by-side amplification reaction that occurs under isothermal conditions on short single-stranded DNA templates using Bst exo- DNA polymerase [2] through cycle-like structure formation [3]. The products of multimerization appear as a ladder on electrophoretic gels and represent tandem repeats that correlate with nucleotide sequence of initial template [[1], [2], [3]]. Here, we present data on multimerization using short (51 nucleotides) DNA templates (LTa-LTf) with different nucleotide sequences and corresponding natural and modified primers (F/R primer pairs) with one, two or three internucleosidic phosphates containing 1,3-dimethyl-2-imino-imidazolidine moieties (phosphoryl guanidine (PG) groups). Primers with three options of PG position were designed: near the 3′-end (Fc1/Rc1, Fc4/Rc4 and Fc6/Rc6 pairs), in the middle (Fc2/Rc2, Fc5/Rc5, Fc7/Rc7 and Fc8/Rc8 pairs) and near the 5′-end (Fc3/Rc3 and Fc9/Rc9 pairs) of the primers. In primers containing two or three PG groups, modifications were separated by one nucleoside with the exception of the Fc8/Rc8 pair where PG groups were separated by two and three nucleosides. Table 1 represents time-to-threshold (Tt) values obtained for amplification of linear (LT) and circular (CT) forms of different DNA templates using unmodified primers. Sequences and molecular masses of modified primers are given in Table 2, Table 3 represents time-to-threshold (Tt) values obtained for amplification of linear LTc and circular CTc templates using modified primers. Raw data are provided in Supplementary file.

**Table 1:** The mean Tt (time-to-threshold) values (minutes) for amplification of linear (LT) and circular (CT) DNA templates (unmodified primers were used).

Templates	Nucleotide sequence, 5′→3′	Linear form (LT)	Circular form (CT)
LTa	GTCACGTCAGTCCTGTAGTGCTCAGTGTCGTCGTACAGCCTACATTGCAGA	51.8 ± 7.2	14.9 ± 1.4
LTb	CTCTCTCTCTCGCTGACGTGCTCAGTGTCGTCGTACAGCCTAAGGAGAAGA	56.1 ± 8.4	13.7 ± 1.1
LTc	CCTCTTGCTTTCGCTCTCGTTCTTTACAGAACACAGACGAGAAGAAGACCA	53.4 ± 5.9	15.2 ± 1.5
LTd	AGGAGAAGACTGCTGACGTGCTCAGTGTCGTCGTACAGCCTACTCTTCCTC	154.8 ± 30.5	12.5 ± 1.6
LTe	ATTATTAGACTGCTGACGTGCTCAGTGTCGTCGTACAGCCTACGCTGCCGC	N/A^a^	N/A
LTf	CTGCCGCGACTGCTGACGTGCTCAGTGTCGTCGTACAGCCTACGATTATTA	163.1 ± 24.7	14.3 ± 2.1

a N/A – no amplification occurs.

**Table 2:** Sequences and molecular masses of phosphoryl guanidine oligonucleotides.

Name	Sequence, 5′→3′	[M] calc.	[M] exp.
Fc1	CCTCTTGCTTTCGCTCTCGTTCTp*TT	7578.05	7585.2
Fc2	СCTCTTGCTTTCp*GCTCTCGTTCTTT	7578.05	7583.6
Fc3	Cp*CTCTTGCTTTCGCTCTCGTTCTTT	7578.05	7588.8
Fc4	CCTCTTGCTTTCGCTCTCGTTCp*Tp*TT	7673.2	7683.0
Fc5	CCTCTTGCTTTCp*GCp*TCTCGTTCTTT	7673.2	7687.8
Fc6	СCTCTTGCTTTCGCTCTCGTTp*Cp*Tp*TT	7768.35	7779.1
Fc7	CCTCTTGCTTTCp*Gp*Cp*TCTCGTTCTTT	7768.35	–
Fc8	CCTCTTGCTp*TTCp*GCp*TCTCGTTCTTT	7768.35	–
Fc9	Cp*Cp*Tp*CTTGCTTTCGCTCTCGTTCTTT	7768.35	7779.5
Rc1	TGGTCTTCTTCTCGTCTGTGTTCTp*GT	8002.25	8010.1
Rc2	TGGTCTTCTTCTCp*GTCTGTGTTCTGT	8002.25	8017.2
Rc3	Tp*GGTCTTCTTCTCGTCTGTGTTCTGT	8002.25	8006.6
Rc4	TGGTCTTCTTCTCGTCTGTGTTCp*Tp*GT	8097.4	8104.8
Rc5	TGGTCTTCTTCTCp*GTp*CTGTGTTCTGT	8097.4	8111.6
Rc6	TGGTCTTCTTCTCGTCTGTGTTp*Cp*Tp*GT	8192.55	8201.2
Rc7	TGGTCTTCTTCTCp*Gp*Tp*CTGTGTTCTGT	8192.55	8207.5
Rc8	TGGTCTTCTTp*CTCp*GTp*CTGTGTTCTGT	8192.55	8198.4
Rc9	Tp*Gp*Gp*TCTTCTTCTCGTCTGTGTTCTGT	8192.55	8204.4

p* corresponds to modified phosphates (PG groups).

**Table 3:** The mean Tt (time-to-threshold) values for amplification of linear LTc and circular CTc DNA templates using phosphoryl guanidine primers (minutes).^a^

	Fc	Fc1	Fc2	Fc3	Fc4	Fc5	Fc6	Fc7	Fc8	Fc9
**Linear template (LTc)**										
Rc	53.4 ± 5.9	50.7 ± 4.4	52.1 ± 6.4	49.4 ± 7.2	N/A^a^	63.5 ± 7.8	N/A	77.1 ± 11.3	53.9 ± 5.3	50.6 ± 4.7
Rc1	43.5 ± 7.4	67.8 ± 5.3	53.2 ± 7.8	47.1 ± 5.8	N/A	67.6 ± 10.5	N/A	74.8 ± 7.3	46.8 ± 7.5	51.7 ± 5.9
Rc2	53.8 ± 8.1	58.5 ± 6.7	50.6 ± 7.1	50.0 ± 10.3	121.2 ± 27.1	69.2 ± 11.8	N/A	80.5 ± 10.6	60.1 ± 9.4	52.7 ± 13.7
Rc3	49.4 ± 9.3	51.2 ± 5.7	51.6 ± 6.9	53.2 ± 6.8	95.7 ± 13.6	62.5 ± 9.4	N/A	76.6 ± 8.1	56.5 ± 10.1	56.3 ± 9.2
Rc4	N/A	N/A	98.6 ± 12.5	87.4 ± 8.2	110.3 ± 14.7	N/A	N/A	N/A	N/A	126.2 ± 34.9
Rc5	67.8 ± 8.6	63.7 ± 5.5	71.0 ± 6.9	68.3 ± 9.2	74.5 ± 5.1	50.8 ± 9.6	N/A	N/A	N/A	61.8 ± 18.3
Rc6	N/A	N/A	N/A	N/A	N/A	N/A	N/A	N/A	N/A	N/A
Rc7	63.4 ± 5.3	77.3 ± 8.2	66.9 ± 10.5	60.4 ± 21.6	63.3 ± 4.8	N/A	N/A	N/A	N/A	66.4 ± 21.5
Rc8	60.5 ± 9.6	70.6 ± 12.8	50.9 ± 12.3	57.9 ± 10.7	N/A	54.6 ± 12.6	N/A	137.1 ± 13.5	47.2 ± 6.3	59.7 ± 14.6
Rc9	51.5 ± 7.7	55.7 ± 6.6	54.5 ± 6.8	46.7 ± 17.5	70.2 ± 19.1	52.4 ± 9.8	N/A	67.9 ± 15.3	56.2 ± 11.4	60.2 ± 5.6
**Circular template (CTc)**										
Rc	15.2 ± 1.5	13.4 ± 1.4	14.6 ± 1.6	15.1 ± 2.2	50.6 ± 1.5	13.2 ± 1.9	N/A	53.6 ± 1.4	13.8 ± 0.9	14.3 ± 1.4
Rc1	17.3 ± 1.8	27.4 ± 2.1	16.5 ± 1.6	15.4 ± 2.1	33.5 ± 1.6	17.7 ± 1.5	N/A	53.5 ± 2.0	17.5 ± 1.4	13.7 ± 1.1
Rc2	14.5 ± 1.7	13.1 ± 1.2	13.5 ± 1.4	14.6 ± 1.5	44.5 ± 1.7	15.8 ± 1.3	N/A	34.6 ± 1.3	14.3 ± 1.5	15.1 ± 1.6
Rc3	15.8 ± 1.1	14.1 ± 1.2	18.3 ± 1.7	15.7 ± 1.8	40.8 ± 1.3	15.7 ± 1.5	N/A	23.6 ± 1.5	14.6 ± 1.6	15.0 ± 1.5
Rc4	60.5 ± 2.2	36.6 ± 1.6	44.7 ± 2.5	49.4 ± 2.0	60.4 ± 1.7	27.4 ± 1.5	N/A	60.2 ± 2.1	71.5 ± 2.7	44.3 ± 1.9
Rc5	47.5 ± 1.7	27.2 ± 1.4	35.2 ± 2.0	25.2 ± 1.2	33.6 ± 1.8	13.9 ± 1.6	N/A	29.7 ± 1.4	34.4 ± 1.5	25.5 ± 1.3
Rc6	N/A	N/A	N/A	N/A	N/A	N/A	N/A	N/A	N/A	N/A
Rc7	53.4 ± 1.4	60.4 ± 1.9	54.8 ± 3.3	55.3 ± 1.4	50.5 ± 1.1	86.4 ± 2.5	N/A	11.6 ± 1.5	15.0 ± 1.5	33.3 ± 1.5
Rc8	15.6 ± 0.9	15.6 ± 1.7	16.5 ± 1.4	15.4 ± 1.2	46.2 ± 2.0	15.4 ± 1.3	N/A	17.8 ± 1.0	44.2 ± 1.8	17.5 ± 1.6
Rc9	13.7 ± 2.5	15.5 ± 2.0	14.8 ± 1.2	15.1 ± 1.2	38.7 ± 1.1	23.7 ± 2.6	N/A	19.1 ± 1.5	21.0 ± 1.7	11.2 ± 1.3

a N/A – no amplification occurs.

## 2 Experimental design, materials, and methods

### 2.1 Materials

The following reagents were used: Bst 2.0 DNA polymerase and Isothermal buffer (New England Biolabs); T4 DNA ligase, exonuclease I, T4 polynucleotide kinase, dNTP (Thermofisher Scientific); SYBR Green I (Lumiprobe); tetrahydrofuran for DNA synthesis (Panreac); 2-cyanoethyl deoxynucleoside phosphoramidites and CPG solid supports for DNA synthesis (Glen Research). All solutions were prepared with highly purified water (>18 MOm) (Millipore).

### 2.2 Oligonucleotides

Linear DNA templates LTa-LTf, unmodified oligonucleotide primers Fa-Ff and Ra-Rf and splint probes Sa-Sf were designed using an OligoAnalyzer tool (Integrated DNA Technologies) and purchased from Syntol (Russia). Oligonucleotides Fc1-Fc9 and Rc1-Rc9 with internucleosidic phosphoryl 1,3-dimethyl-2-imino-imidazolidine groups (phosphoryl guanidine oligonucleotides (PGO)) were synthesized as described in Refs. [4,5]. PGO were isolated by reverse-phase HPLC on an Agilent 1200 HPLC system (USA) using a Zorbax SB-C18 (4.6 × 150 mm) column with a linear gradient of elution buffer (0–50% acetonitrile in 20 mM triethylammonium acetate, pH 7.0, flow rate 2 ml/min). Purified oligonucleotides were concentrated followed by precipitation with 2% LiClO_4_ in acetone, washing with pure acetone and desiccation under vacuum. PGO structures were confirmed by MALDI-TOF mass spectra recorded on a Reflex III Autoflex mass spectrometer (Bruker) using 3-hydroxypicolinic acid or LC-MS/MS ESI MS on an Agilent G6410A mass spectrometer (USA) in a negative ion mode. Molecular masses of phosphoryl guanidine oligonucleotides were calculated using experimental *m*/*z* values. Stock solutions of oligonucleotides were prepared by dilution of precipitates in deionized water. Sequences of all oligonucleotides are given in Ref. [1].

### 2.3 DNA circularization

The circular DNA templates was prepared as follows. One pmol of each linear oligonucleotides LTa-LTf was routinely phosphorylated by T4 polynucleotide kinase in a 10 μl reaction mixture. Then, 5 pmol of corresponding splint probe Sa-Sf and 2 μl of T4 DNA ligase buffer were added, and the reaction mixtures were put in T100 thermal cycler (Bio-Rad Laboratories) for DNA strands annealing. The temperature was slowly decreased from 80 to 25°С within 1 hour. After the end of annealing, 2 μl of 10 mM ATP and 5 U of T4 DNA ligase were added. The mixtures were incubated for 18 h at 8°С, after which the ligase was inactivated at 75°С for 15 min. Then, 1 U of exonuclease I was added in each sample, and the reaction mixtures were incubated for 2 h at 37°С and then for 1 h at 45°С followed by enzyme inactivation at 85°С for 15 min. The circular DNA templates were diluted up to 10^7^ molecules/ul and used for further amplification reactions without additional purification.

### 2.4 Isothermal DNA amplification

All amplification samples were prepared in an UVC/T-M-AR PCR box (Biosan). The working space, dispensers, and plastic ware were preliminarily irradiated with ultraviolet for 20 min. Amplification was carried out in an iQ5 thermal cycler (Bio-Rad Laboratories) in 10 μl of reaction mixture containing 10^7^ linear or 10^7^ circular DNA target copies per reaction, 5 pmol of each primer, 1 μl of 2.5 mM dNTP, 1 × Isothermal buffer, 0.1 × SYBR Green I intercalating dye and 1.5 U of Bst 2.0 polymerase. Each sample was represented in three repeats. The program of amplification consisted of the following steps: 1) 70°С – 30 s, 2) 60°С – 3 h.
