# Supplementary material for: Data on multimerization efficiency for short linear DNA templates and phosphoryl guanidine primers during isothermal amplification with Bst exo- DNA polymerase
Source: Data Brief. 2020 Jan 25;29:105188. doi: 10.1016/j.dib.2020.105188 (PMC7011048; doi:10.1016/j.dib.2020.105188)
Supplement: Multimedia component 1 [file mmc1.docx]

**SUPPLEMENTARY MATERIALS**

**on article**

**Data on multimerization efficiency for short linear DNA templates and phosphoryl guanidine primers during isothermal amplification with Bst exo- DNA polymerase**

**Authors**

Ravil R. Garafutdinov^1^, Assol R. Sakhabutdinova^1^, Maxim S. Kupryushkin^2^, Dmitrii V. Pyshnyi^2^

**Table S1. Raw Ct (cycle threshold) values for amplification of linear (LT) and circular (CT) DNA templates (unmodified primers were used).**

| Templates | Linear form (LT) | Circular form (CT) |
| --- | --- | --- |
| LTa | 110  175  182 | 38  43  53 |
| LTb | 123  167  215 | 35  42  47 |
| LTc | 124  175  182 | 37  47  53 |
| LTd | 272  552  570 | 29  38  46 |
| LTe | N/A* | N/A |
| LTf | 335  556  580 | 33  40  56 |

* N/A - no amplification occurs.

**Figure S1. Typical LC-MS/MS ESI MS spectra of phosphoryl guanidine oligonucleotides bearing one modified phosphate (ESI MS analysis applying negative ions registration mode (non-deconvoluted data)). p* corresponds to modified phosphate groups.**

Fс1 oligonucleotide (5'-CCTCTTGCTTTCGCTCTCGTTCTp*TT-3')


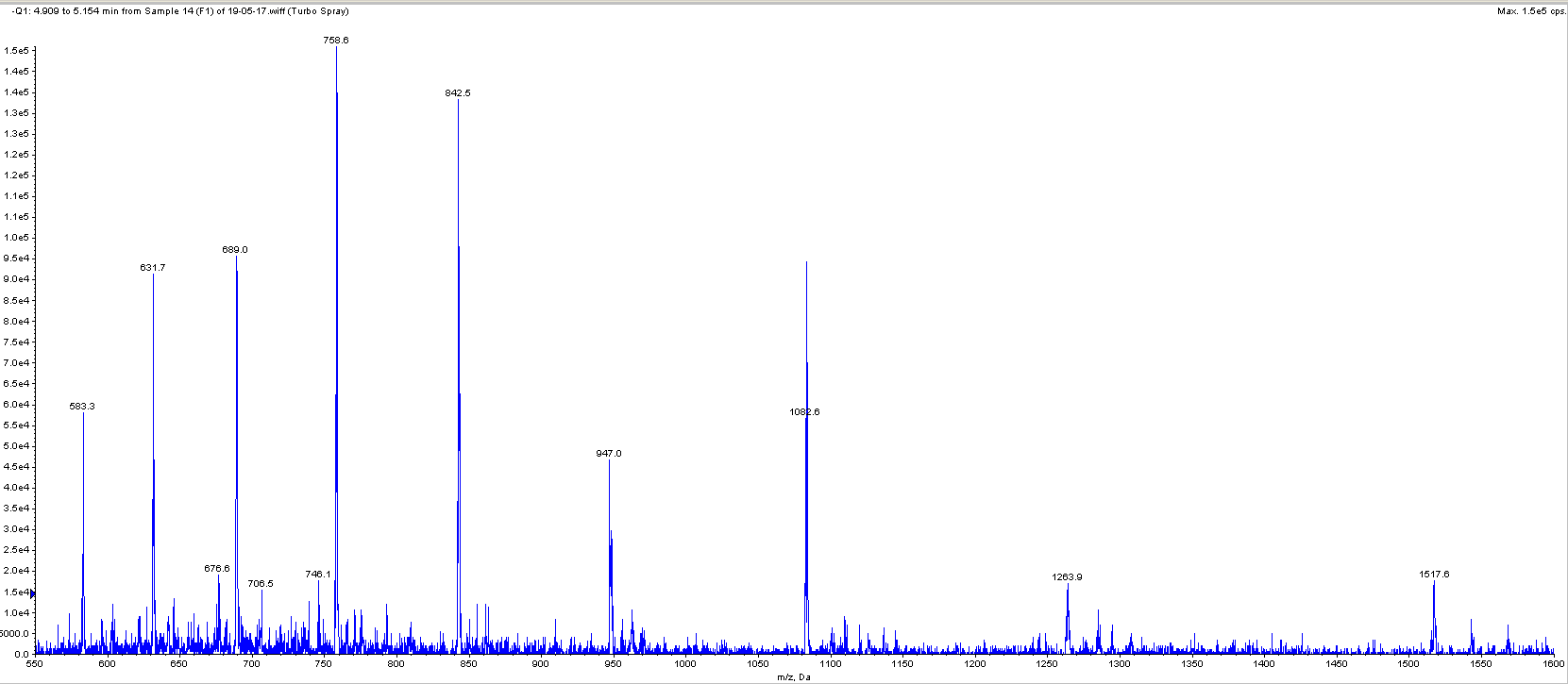


Molecular mass calculated: 7578,05

Molecular mass found: 7585,2

Rс1 oligonucleotide (5'-TGGTCTTCTTCTCGTCTGTGTTCTp*GT-3')


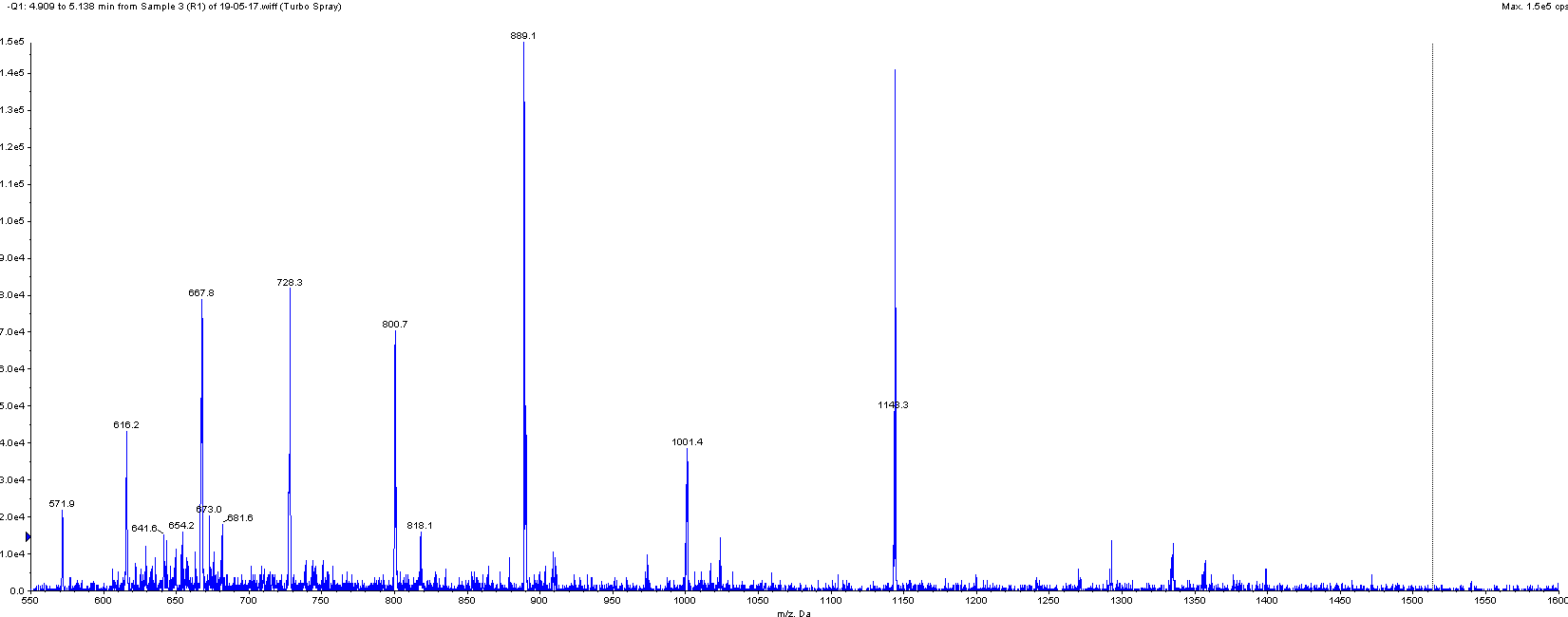


Molecular mass calculated: 8002,25

Molecular mass found: 8010,1**Figure S2. Typical LC-MS/MS ESI MS spectra of phosphoryl guanidine oligonucleotides bearing two modified phosphates (ESI MS analysis applying negative ions registration mode (non-deconvoluted data)). p* corresponds to modified phosphate groups.**

Fс4 oligonucleotide (5'-CCTCTTGCTTTCGCTCTCGTTCp*Tp*TT-3')


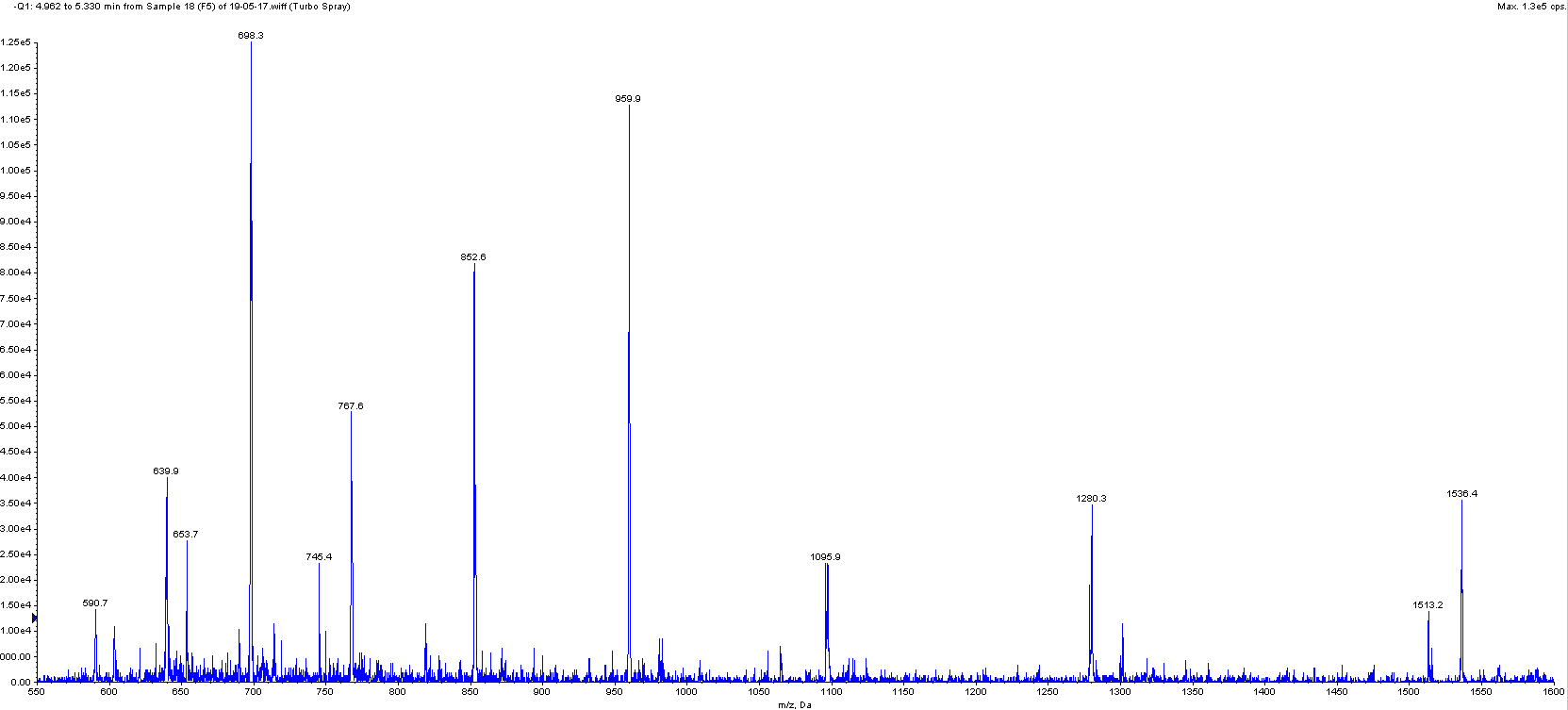


Molecular mass calculated: 7673,2

Molecular mass found: 7683,0

Fс5 oligonucleotide (5'-CCTCTTGCTTTCp*GCp*TCTCGTTCTTT-3')


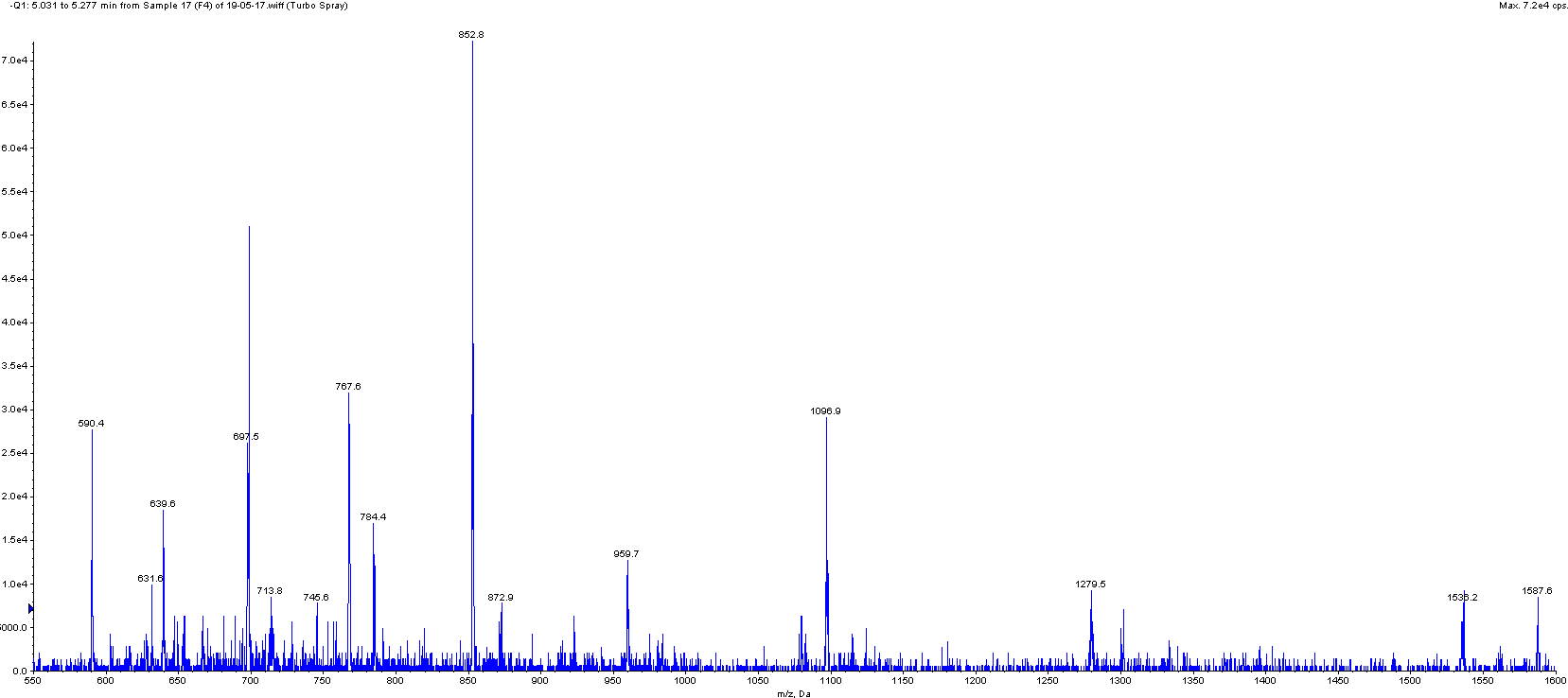


Molecular mass calculated: 7673,2

Molecular mass found: 7687,8

**Figure S3. Typical LC-MS/MS ESI MS spectra of phosphoryl guanidine oligonucleotides bearing three modified phosphates (ESI MS analysis applying negative ions registration mode (non-deconvoluted data)). p* corresponds to modified phosphate groups.**

Fс6 oligonucleotide (5'-СCTCTTGCTTTCGCTCTCGTTp*Cp*Tp*TT-3')


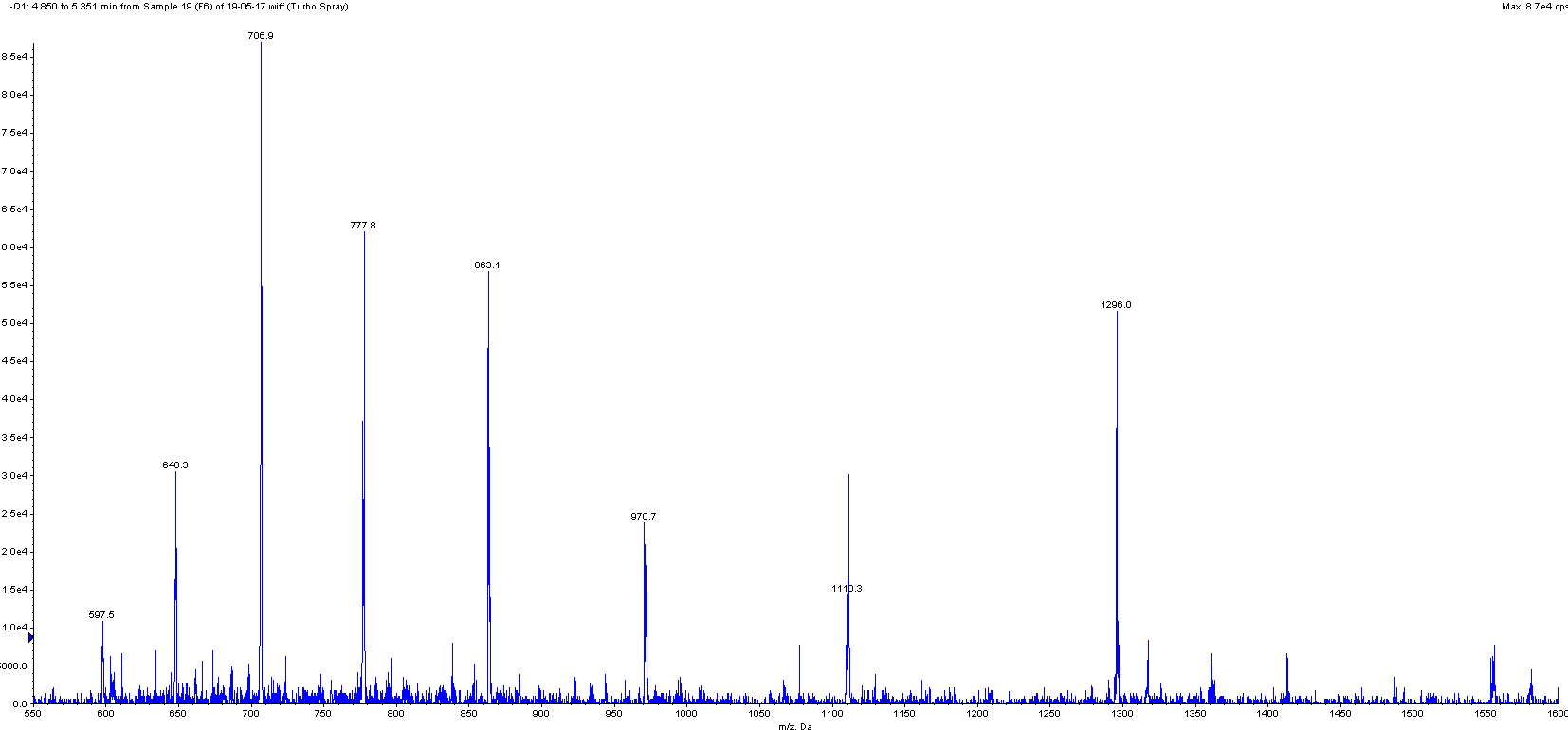


Molecular mass calculated: 7768,35

Molecular mass found: 7779,1

Rс6 oligonucleotide (5'-TGGTCTTCTTCTCGTCTGTGTTp*Cp*Tp*GT-3')


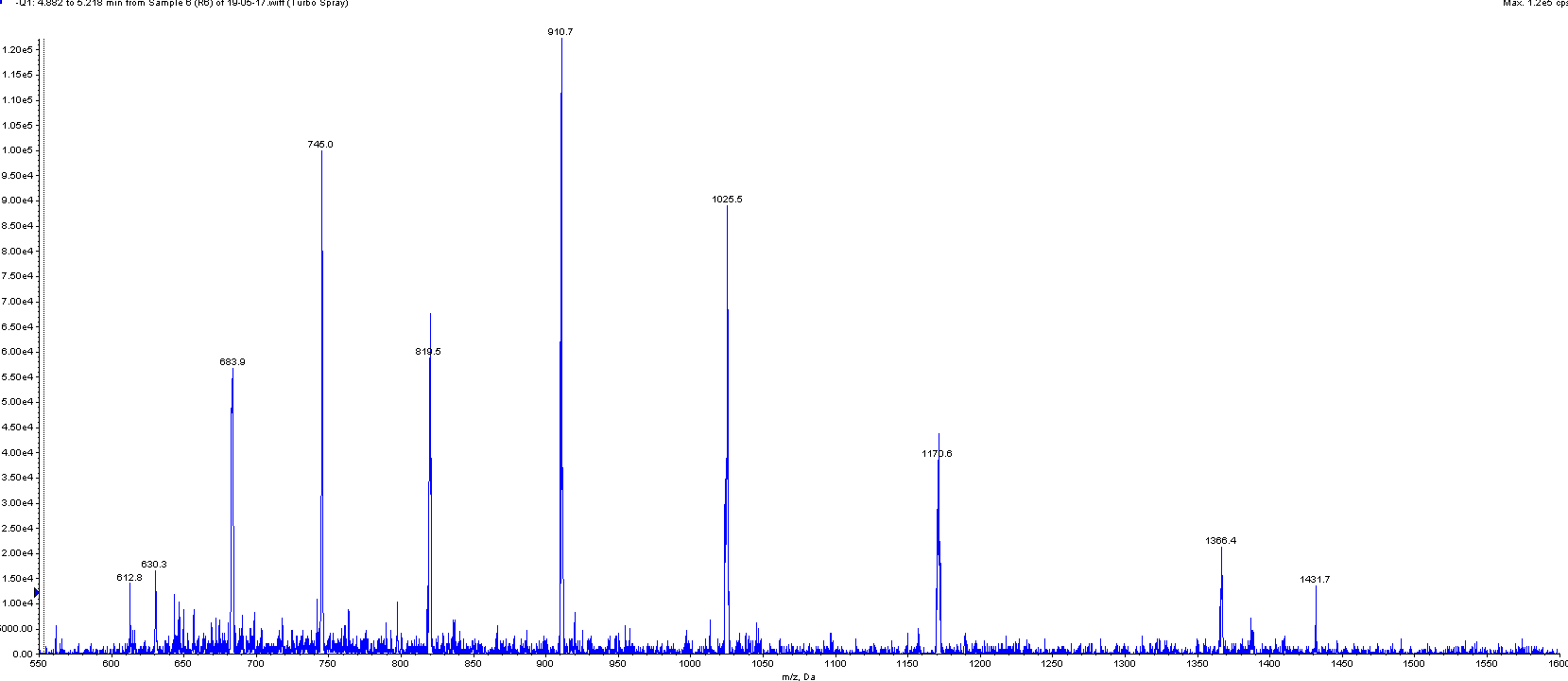


Molecular mass calculated: 8192,55

Molecular mass found: 8201,2

**Table S3. Raw Ct (cycle threshold) values for amplification of linear LTc and circular CTc DNA templates using phosphoryl guanidine primers.**

| **Linear template (LTc)** | | | | | | | | | | |
| --- | --- | --- | --- | --- | --- | --- | --- | --- | --- | --- |
|  | **Fc** | **Fc1** | **Fc2** | **Fc3** | **Fc4** | **Fc5** | **Fc6** | **Fc7** | **Fc8** | **Fc9** |
| **Rc** | 124  175  182 | 132  145  179 | 133  139  197 | 115  138  192 | N/A* | 149  189  234 | N/A | 178  217  299 | 134  159  192 | 127  150  178 |
| **Rc1** | 102  113  177 | 176  200  234 | 122  151  206 | 114  134  176 | N/A | 140  216  252 | N/A | 181  233  259 | 109  125  187 | 124  152  189 |
| **Rc2** | 113  170  201 | 138  178  211 | 121  139  195 | 112  123  215 | 194  427  470 | 153  191  279 | N/A | 178  255  292 | 140  163  238 | 109  121  244 |
| **Rc3** | 105  135  204 | 123  153  185 | 127  140  198 | 126  153  200 | 202  323  337 | 149  168  246 | N/A | 189  223  277 | 124  154  231 | 122  163  222 |
| **Rc4** | N/A | N/A | 225  302  361 | 221  256  310 | 244  347  402 | N/A | N/A | N/A | N/A | 167  432  537 |
| **Rc5** | 151  220  239 | 166  183  224 | 180  205  254 | 151  214  251 | 195  225  251 | 108  139  210 | N/A | N/A | N/A | 114  142  300 |
| **Rc6** | N/A | N/A | N/A | N/A | N/A | N/A | N/A | N/A | N/A | N/A |
| **Rc7** | 157  202  212 | 186  234  276 | 135  226  241 | 90  139  315 | 166  186  218 | N/A | N/A | N/A | N/A | 90  184  324 |
| **Rc8** | 144  159  242 | 141  213  281 | 110  118  230 | 124  159  238 | N/A | 106  145  240 | N/A | 355  384  495 | 116  128  181 | 111  159  267 |
| **Rc9** | 121  140  202 | 139  154  208 | 139  146  207 | 79  91  250 | 120  187  325 | 111  145  216 | N/A | 125  194  292 | 117  151  238 | 147  188  207 |
| **Circular template (CTc)** | | | | | | | | | | |
|  | **Fc** | **Fc1** | **Fc2** | **Fc3** | **Fc4** | **Fc5** | **Fc6** | **Fc7** | **Fc8** | **Fc9** |
| **Rc** | 37  47  53 | 35  37  49 | 35  44  52 | 32  49  55 | 145  149  161 | 31  37  51 | N/A | 152  163  167 | 36  43  45 | 36  42  51 |
| **Rc1** | 41  55  60 | 71  82  94 | 42  48  59 | 37  43  59 | 92  100  110 | 44  55  60 | N/A | 150  160  172 | 44  55  59 | 36  39  48 |
| **Rc2** | 33  48  50 | 35  36  47 | 33  41  48 | 35  45  51 | 124  134  143 | 40  48  54 | N/A | 97  103  111 | 36  41  52 | 36  47  53 |
| **Rc3** | 43  45  54 | 36  42  48 | 47  53  65 | 37  47  57 | 116  121  130 | 38  49  54 | N/A | 62  72  78 | 36  42  53 | 38  43  54 |
| **Rc4** | 170  181  194 | 102  108  119 | 123  130  149 | 140  144  161 | 171  184  185 | 74  83  90 | N/A | 168  183  191 | 198  221  225 | 126  128  145 |
| **Rc5** | 132  147  149 | 75  80  90 | 94  108  115 | 70  74  83 | 91  100  111 | 36  37  52 | N/A | 80  93  94 | 96  102  112 | 70  76  84 |
| **Rc6** | N/A | N/A | N/A | N/A | N/A | N/A | N/A | N/A | N/A | N/A |
| **Rc7** | 154  158  169 | 170  184  190 | 148  161  184 | 158  167  173 | 146  151  158 | 244  263  271 | N/A | 26  36  42 | 36  47  52 | 92  100  108 |
| **Rc8** | 42  46  52 | 39  44  57 | 43  48  58 | 39  48  52 | 126  145  145 | 40  45  54 | N/A | 48  53  59 | 124  131  143 | 46  49  63 |
| **Rc9** | 28  40  55 | 38  43  59 | 38  44  51 | 39  45  52 | 112  113  123 | 55  77  81 | N/A | 49  58  65 | 53  64  72 | 26  35  40 |

* N/A - no amplification occurs.
